# Suppressing Effect of Na^+^/Ca^2+^ Exchanger (NCX) Inhibitors on the Growth of Melanoma Cells

**DOI:** 10.3390/ijms23020901

**Published:** 2022-01-14

**Authors:** Zikai Liu, Qing Cheng, Xiaoli Ma, Mingke Song

**Affiliations:** Department of Pharmacology and Chemical Biology, Institute of Medical Sciences, Shanghai Jiao Tong University School of Medicine, 280 South Chongqing Road, Shanghai 200025, China; 17853509899@163.com (Z.L.); 17865562003@163.com (Q.C.); mxl99@outlook.com (X.M.)

**Keywords:** calcium ions (Ca^2+^), melanoma, Na^+^/Ca^2+^ exchanger, NCX inhibitors, cell death, drug target, Ca^2+^ homeostasis, vemurafenib, melanocytes, pharmacology

## Abstract

The role of calcium ion (Ca^2+^) signaling in tumorigenicity has received increasing attention in melanoma research. Previous Ca^2+^ signaling studies focused on Ca^2+^ entry routes, but rarely explored the role of Ca^2+^ extrusion. Functioning of the Na^+^/Ca^2+^ exchanger (NCX) on the plasma membrane is the major way of Ca^2+^ extrusion, but very few associations between NCX and melanoma have been reported. Here, we explored whether pharmacological modulation of the NCX could suppress melanoma and promise new therapeutic strategies. Methods included cell viability assay, Ca^2+^ imaging, immunoblotting, and cell death analysis. The NCX inhibitors SN-6 and YM-244769 were used to selectively block reverse operation of the NCX. Bepridil, KB-R7943, and CB-DMB blocked either reverse or forward NCX operation. We found that blocking the reverse NCX with SN-6 or YM-244769 (5–100 μM) did not affect melanoma cells or increase cytosolic Ca^2+^. Bepridil, KB-R7943, and CB-DMB all significantly suppressed melanoma cells with IC_50_ values of 3–20 μM. Bepridil and KB-R7943 elevated intracellular Ca^2+^ level of melanoma. Bepridil-induced melanoma cell death came from cell cycle arrest and enhanced apoptosis, which were all attenuated by the Ca^2+^ chelator BAPTA-AM. As compared with melanoma, normal melanocytes had lower NCX1 expression and were less sensitive to the cytotoxicity of bepridil. In conclusion, blockade of the forward but not the reverse NCX leads to Ca^2+^-related cell death in melanoma and the NCX is a potential drug target for cancer therapy.

## 1. Introduction

Melanoma, one of the most common skin malignancies, accounts for 1–3% of all malignant tumors [1,2]. The age-adjusted incidence rate of melanoma worldwide has increased by up to 6% per annum; this rising rate makes it one of the fastest growing cancers [2,3,4,5,6,7,8]. Melanomas have high frequency of mutations that include mutant BRAF, mutant NRAS, mutant neurofibromatosis type 1 (NF1), and the triple wild type [9]. Although BRAF and mitogen-activated protein kinase (MEK) inhibitors has improved current situation of melanoma therapy, challenges still remain regarding how to overcome drug resistance and meet the demand of more effective targeted therapies [10].

The calcium ion (Ca^2+^), one of the ubiquitous intracellular second messengers, plays critical roles in cell division, growth, differentiation, apoptosis, and necrosis [11]. Recently, modulation of Ca^2+^ signaling is receiving increasing attention in the area of cancer research because Ca^2+^ participates in tumorigenicity, metastasis, and invasion of cancer cells [12,13]. Human melanoma cells express voltage-gated Ca^2+^ channels (VGCCs) from the families of the Ca_v_1, Ca_v_2, and Ca_v_3 (T-type) [14]. The Ca_v_3 isoforms was reported to promote progression of melanoma cell cycle and blockade of T-type channels increased apoptosis in malignant melanoma [14,15]. The store-operated Ca^2+^ entry (SOCE) was found to regulate proliferation of melanoma cells and SOCE-mediated Ca^2+^ influx likely endowed some melanoma cells with apoptotic resistance [16,17]. The transient receptor potential melastatin (TRPM) channels such as TPRM8, TPRM2, and TPRM7 favor tumor progression through overexpression and hyperfunction in melanoma [18]. Overall, previous melanoma Ca^2+^ signaling studies focused on Ca^2+^ entry and its molecular machinery, but rarely explored the role of Ca^2+^ extrusion mechanisms in tumor growth.

Cellular Ca^2+^ homeostasis is maintained by Ca^2+^ channels/transporters that control Ca^2+^ influx and efflux [13]. The extrusion of cytosolic Ca^2+^ is through functioning activities of the plasma membrane Ca^2+^-ATPase pump (PMCA pump) and the Na^+^/Ca^2+^ exchanger (NCX) [19]. Ca^2+^ transporting capacity of the NCX is routinely up to 60-fold higher than that of PMCA; therefore, activity of the NCX is the major way of Ca^2+^ extrusion when cytosolic free Ca^2+^ goes over resting level, particularly in the cardiac and neuronal cells [20]. Altered expression of NCX isoforms (NCX1, NCX2 and NCX3) have been largely identified in the ischemic and degenerative brain injuries and NCX dysfunction is implicated in many nervous system diseases [21]. Melanomas originates from melanocytes, which are derived from neural crest cells or emerge by detaching from the nerves that innervate the skin [22]. However, very few association between NCX and human melanoma has been reported either in pharmacology or in tumor biology studies [23,24], except one recent work which was performed to detect NCX1 expression in two melanoma cell lines and tested the effect of NCX inhibitors [25].

The NCX located on cell membrane is a bi-directional transporter that can import three Na^+^ into cytoplasm in exchange for one Ca^2+^ exit (the forward mode) or drive three Na^+^ efflux for one Ca^2+^ uptake (the reverse mode) [26]. The working direction of NCXs is determined by the electrochemical gradients for Ca^2+^ and Na^+^ as well as cell membrane potential [27]. Many factors such as pH, ATP, level of nitric oxide (NO), and phosphatidylinositol 4,5-bisphosphate (PIP2) may influence the direction of NCX operation [23,25,28,29,30,31]. The NCX inhibitors bepridil and CB-DMB block both the forward and reverse mode of NCX, whereas KB-R7943 inhibits the reverse NCX more potently than its forward mode [32,33]. SN-6 and YM-244769 are inhibitors that preferentially block the reverse mode of NCX [30,32]. Previously, the NCX inhibitors were heavily used as pharmacological tools to study the role of NCXs in the hypoxic/ischemic brain, heart, and renal injury [21]. Upon these pathological models, some NCX inhibitors exerted a protective effect, but others appeared to exacerbate such injury [21,34].

We here envisioned that the blocking action of NCX inhibitors may interfere with intracellular Ca^2+^ homeostasis of melanoma cells, which in turn may affect tumor cell survival. To test this speculation, we performed pharmacological experiments on BRAF and NRAS mutated human melanoma cells with above mentioned NCX inhibitors, and explored whether modulation of the NCX can promise new therapeutic strategies.

## 2. Results

### 2.1. Effect of NCX Blockers on Viability of Melanoma Cells

**Figure 1:**
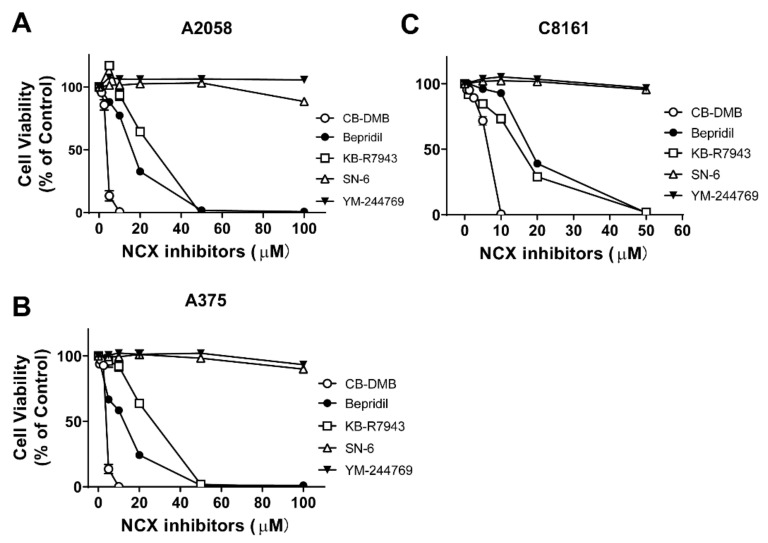
Effect of NCX inhibitors on viability of melanoma cells. (**A**–**C**) Viability of BRAF mutated human melanoma cell lines (A2058, A375, and C8161) after exposed to NCX blockers bepridil, KB-R7943, CB-DMB, SN-6, or YM-244769 at 0–100 µM for 48 h. *n* = 4–5 independent tests per group.

Concentrations of NCX inhibitors bepridil and KB-R7943 to exert half-maximal inhibition (IC_50_) of the NCX were reported to be around 14–25 μM [32]. The IC_50_ values of CB-DMB were in the nanomolar range for the NCX; the IC_50_ values of SN-6 and YM-244769 were ~4 μM [35]. We treated BRAF-mutated human melanoma cell lines A2058, A375, and C8161, respectively, with bepridil, CB-DMB, KB-R7943, SN-6, or YM-244769 for 48 h. Cell viability of each group was analyzed with the CCK-8 assay method. The final concentrations of NCX blockers dissolved in cell culture medium were from 0.625 to 100 μM. The dose-response relationship experiment revealed that 2.5, 5.0, and 10 μM CB-DMB suppressed viability of melanoma cells to 75.2–85.3%, 6.5–22.7%, and 0.35–1.40% of control cells (Figure 1A–C). Bepridil at 10, 20, and 50 μM suppressed viability of melanoma cells to 76.5–89.7%, 22.7–43.8%, and 0.54–2.65% of control cells, respectively. KB-R7943 at 10, 20, and 50 μM suppressed melanoma cells to 71.2–83.5%, 25.8–56.5%, and 0.71–2.64% of control level. By contrast, SN-6 or YM-244769 did not appear to affect melanoma cell proliferation; viability of A2058, A375, and C8161 melanoma cells were at 92.3–108.4% of control level after the 48-h incubation with SN-6 or YM-244769 (5–50 μM). Table 1 shows IC_50_ values of bepridil, CB-DMB and KB-R7943 that inhibited the growth of A2058, A375 and C8161 cells. We also used a hydrogel-based three-dimensional (3D) culture platform to culture A2058 cells and tested their response to the NCX blocker bepridil. We found that viability of A2058 cells in this 3D culture were reduced to 50% of vehicle-treated control level (Supplementary Figure S1) after incubation with bepridil (25 μM, 48 h).

### 2.2. Effect of NCX Inhibitors on Cytosolic Ca^2+^ Level in Melanoma Cells

**Figure 2:**
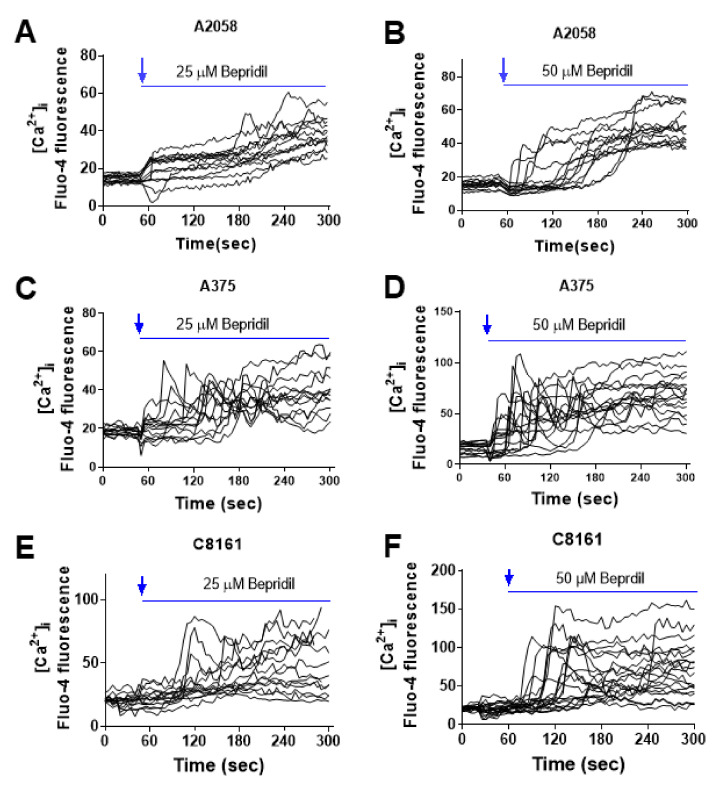
Effect of the NCX blocker bepridil on the level of cytosolic Ca^2+^ ([Ca^2+^]_i_) measured by Ca^2+^ imaging assay. (**A**,**B**) Flua-4 AM loaded A2058 melanoma cells were imaged and perfused with bepridil (25 or 50 µM) in the solution containing 2 mM Ca^2+^. (**C**,**D**) A375 melanoma cells were loaded with Flua-4 AM, imaged and perfused with bepridil (25 or 50 µM). (**E**,**F**) Ca^2+^ imaging traces of C8161 melanoma cells. The recording traces were representatives of 3 separate experiments, and *n* = 15–20 cells were imaged per experiment.

SN-6 and YM-244769 preferentially inhibit intracellular Na^+^-dependent Ca^2+^ uptake (reverse NCX operation) but cannot affect extracellular Na^+^-dependent Ca^2+^ efflux (forward NCX operation) [30,32,36]. Bepridil and KB-R7943 block both forward and reverse operation of the NCX [32]. Blockade of the forward NCX may disrupt intracellular Ca^2+^ homeostasis and elevate the level of cytosolic Ca^2+^. To test our speculation, we applied bepridil to melanoma cells that were pre-loaded with a Ca^2+^ indicator Fluo-4 in the media containing 2 mM Ca^2+^ and 1% FBS. The Ca^2+^ imaging data from A2058, A375 and C8161 cells show that, shortly after perfusion with the NCX inhibitor bepridil (25, 50 μM), the cytosolic Ca^2+^ signal in melanoma cells began to increase and sustained this elevation over the recording course (Figure 2A–F).

**Figure 3:**
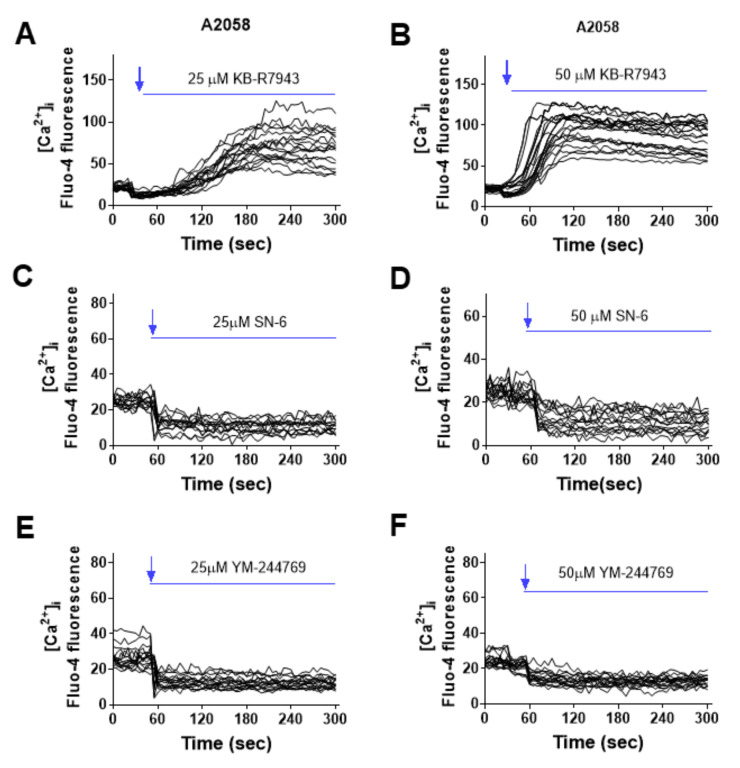
Effect of KB-R7943, SN-6 and YM-244769 on the level of cytosolic Ca^2+^ ([Ca^2+^]_i_) in A2058 melanoma cells. (**A**,**B**) Flua-4 AM loaded A2058 melanoma cells were perfused with KB-R7943 (25 or 50 µM) in the solution containing 2 mM Ca^2+^. KB-R7943 elevated cytosolic Ca^2+^ level. (**C**,**D**) A2058 cells were loaded with Flua-4 AM, imaged and perfused with SN-6 (25 or 50 µM). (**E**,**F**) Ca^2+^ imaging traces of A2058 cells that were perfused with YM-244769 (25 or 50 µM). The recording traces were representatives of 3 separate experiments; *n* = 14–18 cells were imaged per experiment.

Application of KB-R7943 (25, 50 μM) also incurred an elevation of cytosolic Ca^2+^ in A2058 cells that were pre-loaded with Fluo-4 (Figure 3A,B). We then introduced the reverse NCX inhibitors SN-6 and YM-244769 to melanoma cells and examined their effect on intracellular Ca^2+^ content. The Ca^2+^ imaging result showed that SN-6 and YM-244769 (25, 50 μM) did not increase cytosolic Ca^2+^, but caused a certain degree of Ca^2+^ decline in A2058 cells (Figure 3C–F).

### 2.3. Bepridil-Caused Cell Cycle Arrest and Apoptosis in Melanoma Cells

**Figure 4:**
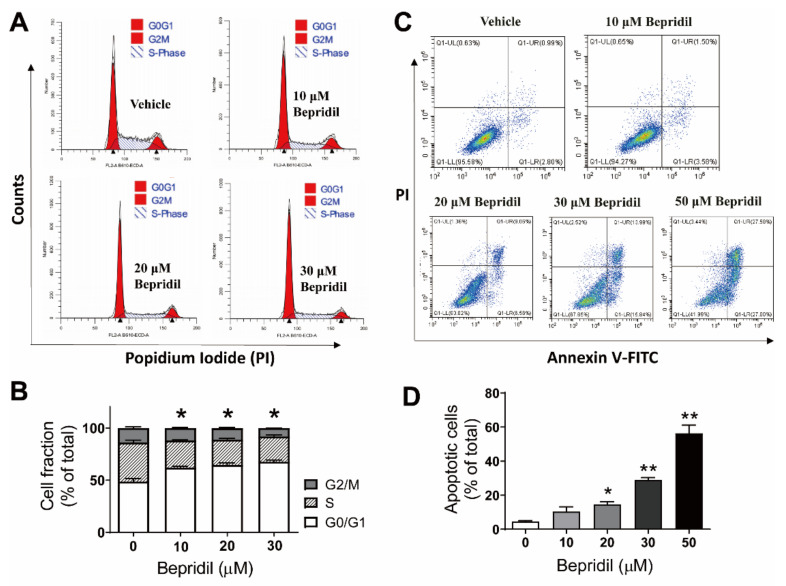
Bepridil-induced cell cycle arrest and apoptosis in melanoma cells. (**A**,**B**) Cell cycle profile of A2058 cells incubated with vehicle or bepridil (10, 20, and 30 μM) for 18 h. The vehicle was cell culture medium containing DMSO (1/1000, *v*/*v*). Bepridil increased the proportion of cells in G0/G1 phase. * *p* < 0.05, G0/G1 phase of bepridil treated groups vs. vehicle treatment, *n* = 5 experiments. (**C**,**D**) A2058 cells were treated with bepridil (10, 20, 30, 50 μM) for 24 h and then analyzed with Annexin V-FITC/PI apoptosis assay. Bepridil increased the fraction of apoptotic cells (Annexin V+). * *p* < 0.05 and ** *p* < 0.01, compared with vehicle treatment; one-way ANOVA followed by a Tukey’s post-hoc test, *n* = 5.

To disclose cell death mechanisms behind bepridil-induced melanoma cytotoxicity, we performed apoptosis and cell cycle analysis. A2058 cell cultures were incubated with bepridil at 10, 20, and 30 μM for 18 h. We found that bepridil increased the cell fraction in G_0_/G_1_ phase of cell cycle and decreased S phase cell proportion (Figure 4A,B). To detect apoptotic cell death, A2058 cells were treated with bepridil (10, 20, 30, 50 μM) for 24 h, then analyzed with Annexin V-FITC/PI staining agent. The result is that bepridil incurred a dose-dependent increase in the fraction of apoptotic (Annexin V^+^) cells (Figure 4C,D).

**Figure 5:**
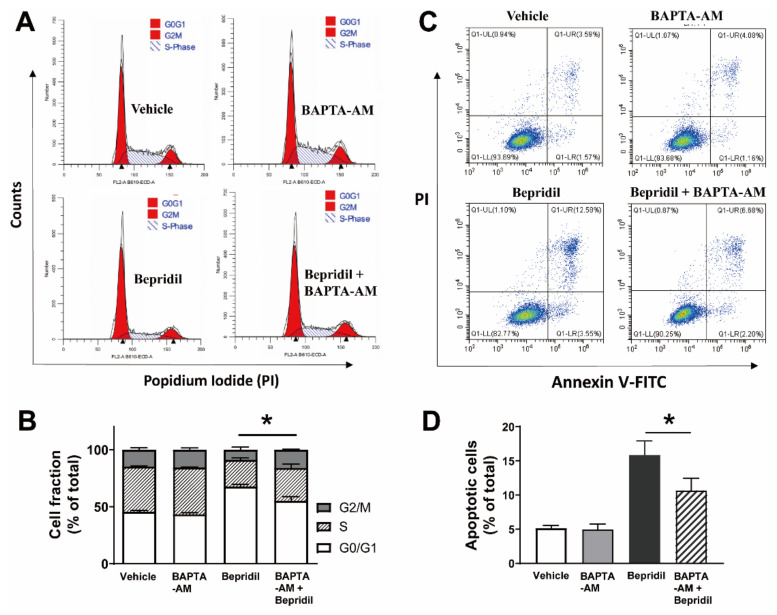
The Ca^2+^ chelator BAPTA-AM prevented bepridil to induce cell cycle arrest and apoptosis. (**A**,**B**) Cell cycle profile of A2058 cells incubated with vehicle, BAPTA-AM (10 μM), bepridil (25 μM), and BAPTA-AM + bepridil for 18 h. BAPTA-AM alone did not incur cell fraction changes but significantly attenuated bepridil-induced cell cycle arrest. * *p* < 0.05, G_0_/G_1_ phase of bepridil vs. BAPTA-AM + bepridil, *n* = 5 independent experiments. (**C**,**D**) A2058 cells treated with vehicle, BAPTA-AM (10 μM), bepridil (25 μM), and BAPTA-AM + bepridil for 24 h. * *p* < 0.05, apoptotic cell fraction in bepridil treatment vs. BAPTA-AM + bepridil; one-way ANOVA followed by a Tukey’s post hoc test, *n* = 5.

Having found that bepridil elevated cytosolic Ca^2+^ content in melanoma cells, we next employed BAPTA-AM to suppress intracellular Ca^2+^ signaling in A2058 cells and examined whether Ca^2+^ was involved in bepridil-induced cell cycle arrest and apoptosis. To do this, melanoma cells were preloaded with a Ca^2+^ chelator BAPTA-AM (10 μM) for 2 h in advance. The result is that 18 h incubation with BAPTA-AM (10 μM) did not change proportion of cells in G_0_/G_1_, S, and G2/M phases while 18-h bepridil (25 μM) incubation markedly increased cell fraction in G_0_/G_1_ phase. This G_0_/G_1_ arresting effect was significantly attenuated if the cells were pre-loaded with BAPTA-AM (Figure 5A,B). Similarly, BAPTA-AM (10 μM) alone for 24 h did not promote A2058 apoptosis, but significantly prevented bepridil to augment apoptosis (Figure 5C,D).

### 2.4. Effect of Bepridil on Melanocytes and NRAS Mutated Melanoma Cells

**Figure 6:**
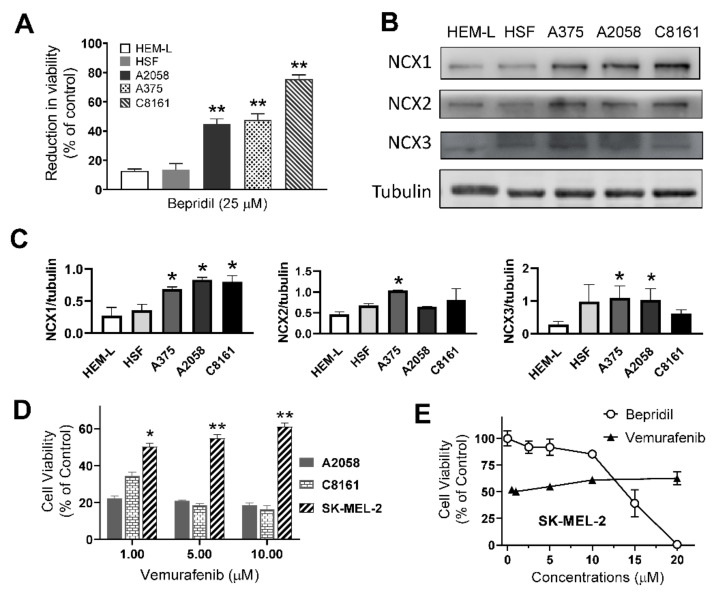
The impact of bepridil on melanocytes and NRAS mutated melanoma cell. (**A**) Human melanoma cells, epidermal melanocytes-light (HEM-L) cells, and skin fibroblasts (HSF) were treated with bepridil (25 μM) for 24 h. Reduction in cell viability was expressed as the percent reduction with respect to control cells. ** *p* < 0.01, each cell line compared with HEM-L, *n* = 3. (**B**) The three NCX isoforms detected in these cell lines. (**C**) Quantifications of the three NCX isoforms obtained by western blotting. * *p* < 0.05, each cell line compared with HEM-L, *n* = 3. (**D**) Effects of the BRAF inhibitor vemurafenib on viability of A2058, C8161 and SK-MEL-2 cells, 72 h. * *p* < 0.05, ** *p* < 0.01, SK-ME-2 compared with C8161 or A2058 cell line; one-way ANOVA followed by a Tukey’s post-hoc test, *n* = 5. (**E**) The dose–response relation between pharmacological agents and viability of SK-MEL-2 cells.

We chose human epidermal melanocytes-light (HEM-L) cells and skin fibroblasts (HSF) as normal cellular counterpart of melanoma and treated them with bepridil (25 μM) for 24 h. We found that A2058, A375 and C8161 melanoma cells were markedly inhibited (43–88% reduction in viability) by 24-h incubation with bepridil; while HEM-L and HSF cells were much less affected by bepridil (11–18% reduction) (Figure 6A). Western blotting analysis of cell samples revealed that they express three NCX isoforms (Supplementary Figure S2); as compared with HEM-L cells, melanoma cells (A2058, A375 and C8161) have higher expression level of the NCX1 isoform (Figure 6B,C). We then detected the NCX1 in other melanoma cell lines including SK-MEL-2, SK-MEL-28, WM-115, and A875 (Supplementary Figure S3A–C). The Western blotting result showed that expression level of the NCX1 in these melanoma cells were significantly greater than that in HEM-L cells (Supplementary Figure S3D). Forty-eight hour treatment with the NCX blocker bepridil dose-dependently suppressed viability of A875, WM-115, and SK-MEL-28 cell lines, yielding IC_50_ values around 1.60, 8.70, and 13.64 μM, respectively (Supplementary Figure S3E).

The SK-MEL-2 cell line has NRAS mutation that is believed to be implicated in acquired resistance to BRAF inhibitors [37]. Herein, we tested the sensitivity of A2058, C8161, and SK-MEL-2 cells to a BRAF inhibitor vemurafenib. BRAF mutated A2058 and C8161 cells were significantly suppressed by vemurafenib (1, 5, 10 μM), while SK-MEL-2 cells displayed low sensitivity to vemurafenib (Figure 6D). We then challenged SK-MEL-2 cells with the NCX inhibitor bepridil (2.5–20 μM) and observed a dose-dependent inhibition with an IC_50_ value of 14.6 μM approximately (Figure 6E). Although 1 μM vemurafenib reduced viability of SK-MEL-2 cells to 50% of control level, this efficacy did not gain further increase even its concentration was elevated to 20 μM. So, bepridil demonstrated better potency and efficacy than vemurafenib in suppression of SK-MEL-2 cells.

## 3. Discussion

Little is known about the role of NCXs in melanoma cells; it is of strong interest to study possible involvement of the NCX and analyze its therapeutic potential in melanoma [38,39]. In the present work, we examined whether and how pharmacological blockade of the NCX could affect cell growth of melanoma. We found that in vitro treatment with bepridil, KB-R7943, or CB-DMB all generated a pronounced suppressing effect on A2058, A375, and C8161 melanoma cells. Concentrations of these NCX blockers to exert half-maximal inhibition of tumor cells were 3 to 20 μM. By contrast, neither SN-6 nor MY-244769 was able to exert influence on melanoma cells in the concentration range of 5 to 100 μM. It is well established that SN-6 or YM244769 selectively blocks reverse mode of the NCX, while bepridil, KB-R7943, or CB-DMB inhibits both forward and reverse operation of the NCX. Therefore, it can be concluded that the tumor suppression effect of bepridil, KB-R7943 and CB-DMB probably resulted from blockade of the forward but not the reverse operation of the NCX.

**Figure 7:**
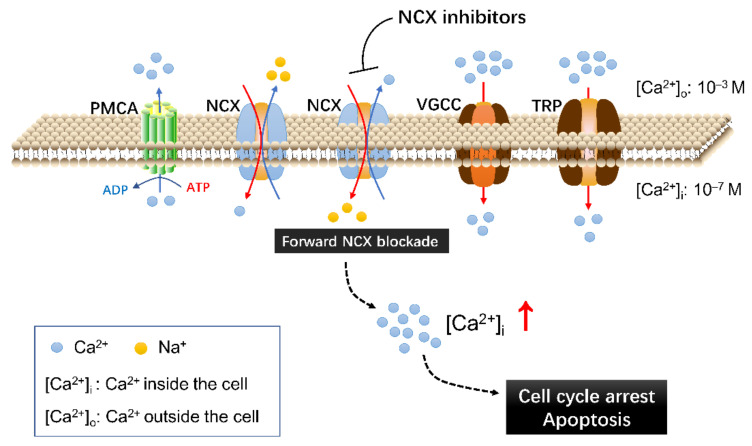
The action of NCX inhibitors on the Ca^2+^ transport/signaling system of melanoma cells and relevant consequences. Blockade of Na^+^-dependent Ca^2+^ efflux (forward NCX operation) by some NCX inhibitors occluded NCX-mediated Ca^2+^ extrusion, while extracellular Ca^2+^ still entered cells through VGCCs, TRP, and other Ca^2+^-permeable channels, resulting in Ca^2+^ accumulation and Ca^2+^-dependent cell death. Ca^2+^: Calcium ions; PMCA, plasma membrane Ca^2+^-ATPase; NCX, Na^+^/Ca^2+^ exchanger; VGCC: voltage-gated Ca^2+^ channel; TRP, transient receptor potential channel; M, mol/L.

This preliminary conclusion was supported by the Ca^2+^ imaging experiment and cell death mechanism delineation. We showed that application of bepridil (25, 50 μM) to A2058, A375 or C8161 cells all incurred a sustained elevation of cytosolic Ca^2+^; similarly, KB-R7943 (25, 50 μM) elevated intracellular Ca^2+^ content in A2058 cells. By contrast, SN-6 or YM244769 selectively inhibited NCX-mediated Ca^2+^ uptake (reverse NCX operation); this blocking action did not increase but reduce Ca^2+^ influx to a certain extent. One can imagine that KB-R7943 or bepridil-caused Ca^2+^ entry was from blockade of Na^+^-dependent Ca^2+^ efflux (forward NCX operation) but not from inhibition of the reverse NCX operation. This blocking action occluded NCX-mediated Ca^2+^ extrusion, while extracellular Ca^2+^ still entered cells through Ca^2+^-permeable VGCCs, SOCE, and TRPM channels, thus leading to a net Ca^2+^ influx. Human melanoma cells release high level of glutamate which activates glutamate receptor and may stimulate additional Ca^2+^ influx [40,41]. Cell death analysis revealed that bepridil incurred a G0/G1 cell cycle arrest and apoptosis in melanoma cells; the Ca^2+^ chelator BAPTA-AM prevented bepridil to promote apoptosis and cell cycle arrest. This implied that bepridil-caused cell cycle and apoptosis was partly mediated by intracellular Ca^2+^ elevation. It has been documented that Ca^2+^ signal directly regulated cell cycle via mitogen-activated protein kinase (MAPK) phosphorylation or through activating immediate early genes in many types of cancer cells [13]. Excessive Ca^2+^ influx could trigger endoplasmic reticulum (ER)-mitochondria Ca^2+^ release, leading to mitochondrial Ca^2+^ accumulation and consequently Ca^2+^-dependent apoptosis [42]. Collectively, we proved that the cytotoxicity of bepridil mostly resulted from blocking the forward NCX and increase of cytosolic Ca^2+^ as illustrated in Figure 7.

Tumor treatment should try to avoid adverse effects on normal cells. In our study, 24-h treatment with bepridil exerted strong suppressing effect on melanoma cells but had relatively weak influence on normal melanocytes and skin fibroblasts. The resistance of melanocytes to bepridil probably because its expression level of NCX1 isoform is lower than that in melanoma cells. Although this point needs to be proved through conducting more verification experiments, its rationality can be supported by the report that melanoma’s sensitivity to NCX inhibitors was associated to an increased NCX1 expression [25]. The NCX of melanoma can switch between forward and reverse operation and this mode exchange might be related to distinct tumor metastatic status [24,43]. Nevertheless, the functioning and importance of NCX operation direction in tumor behaviors still remains to be established [38]. Our in vitro tests demonstrated that blockade of the forward NCX effectively inhibited the growth of melanoma cells which have either BRAF or NRAS mutation. This suggests that the NCX on the plasma membrane of melanoma cells is a potential cancer drug target.

To beat melanoma via targeting the NCX raises another concern that this anti-melanoma strategy will inevitably cause cardiac side effect because the NCX is responsible for extruding Ca^2+^ from cardiomyocytes [44,45]. This issue could be resolved through using pharmacological approaches that specifically disrupt Ca^2+^ homeostasis in melanoma cells. Tyrosinase, an enzyme located in melanosomes, is overexpressed in and relatively specific for malignant melanoma [46,47]. Recently, tyrosinase has been emphasized as a prodrug-converting enzyme for the treatment of melanoma [48,49,50]. The idea is to conjugate NCX inhibitors with substrates of tyrosinase to make specific anti-melanoma prodrugs that can be released by cleavage ability of tyrosinase and induce cell death of melanoma.

## 4. Materials and Methods

### 4.1. Culture of Melanoma Cells and Melanocytes

BRAF-mutated human melanoma cell lines A2058 (Cat# CRL-11147), A375 (Cat# CRL-1619), and C8161 (Cat# XY-XB-2152), SK-MEL-28 (Cat# HTB-72); NRAS-mutated melanoma cell line SK-MEL-2 (Cat# HTB-68); and Human skin fibroblasts (HSF, Cat# CC-Y1274) were all purchased from American Type Culture Collection (Rockville, MD, USA). WM-115 (Cat# CRL-1675) was from Shanghai zeye Bio-Technology Co., Ltd. and A875 (Cat# CL-0255) was from Procell Life Science & Technology Co., Ltd. These cells were cultured in Dulbecco’s Modified Eagle’s Medium (DMEM) (Gibco, Carlsbad, CA, USA) containing L-glutamine and 10% fetal bovine serum (FBS) (Gibco), and maintained no more than 30 passages. SK-MEL-28 cells were cultured in RPMI-1640 +10%FBS. Human epidermal melanocytes-light (HEM-L, Cat# 2200) were purchased from ScienCell Research Laboritories (Carlsbad, CA, USA) and cultured in melanocyte medium supplemented with 0.5% FBS and MelGS growth supplement. Two to ten passages of HEM-L cells were used for our study. All cells were maintained at 37 °C in an incubator with a humidified atmosphere containing 5% CO_2_ and 95% air. Hydrogel-based three-dimensional (3D) cell culture platform was purchased from Kingmorn life science (Shanghai, China). 3D culture of A2058 cells was performed by following the manufacturer’s instructions with the same culture medium as above mentioned.

### 4.2. Reagents

The cell counting kit-8 (CCK8) and Annexin V-FITC/PI apoptosis assay kit were purchased from DOJINDO LABORATORIES (Tokyo, Japan). Bepridil (Cat# 4117), KB-R7943 (Cat# 1244), YM-244769 (Cat# 4544), SN-6 (Cat# 2184), and [1,2-bis(2-aminophenoxy) ethane-N,N,N′,N′-tetraacetic acid (BAPTA-AM)] (Cat# 2787) were from Tocris Bioscience (Minneapolis, USA). The BRAF inhibitor vemurafenib (Cat# HY-12057) were from MedChemExpress (Monmouth Junction, NJ, USA). These compounds were dissolved in dimethyl sulfoxide (DMSO) to make stock solution. Propidium iodide (PI) and Ribonuclease A were from Sigma Aldrich (St. Louis, MO, USA). Cell-permeable Ca^2+^ indicator Fluo-4 AM was from Invitrogen Life Technologies (Waltham, MA, USA). The primary antibody for NCX1 isoform (Cat# ab177952) was from Abcam (Cambridge, MA, USA); anti-NCX2 (Cat# ANX-012) and NCX3 (Cat# ANX-013) antibodies were from Alomone Labs (Jerusalem, Israel). Mouse α-tubulin antibody (Cat# 2144) was from Cell Signaling Technology (Boston, MA, USA).

### 4.3. Cell Viability Assay

Cell viability was assessed by a Cell Counting Kit-8 (DOJINDO, Japan). Briefly, cells were plated in 96-well cell culture plates at 6000 per well and cultured overnight. Cells were treated with test reagents for 24–72 h according to different requirements. Control cells were treated with cell culture medium and assessed at the same time point as corresponding reagents. CCK-8 solution (10 μL) was added to each culture wells and incubated for 30 min at 37 °C. Absorbance at 450 nm was measured using an automatic microplate reader (Molecular Devices, San Jose, CA, USA). The vehicle treatment, i.e., incubation with culture medium containing DMSO (1/1000, *v*/*v*), did not affect cell viability in our system.

### 4.4. Ca^2+^ Imaging Experiment

Cells were cultured in 96-well plates at 6000 per well and loaded with the cell membrane permeable Ca^2+^ dye Fluo-4 AM (5 μM) in HEPES-buffered solution for 40 min. The HEPES solution was supplemented with 1% FBS at pH 7.4. Fluo-4 AM loaded cells was washed completely to remove background and mounted in a Leica TCS SP8 confocal system (Leica Microsystems, Wetzlar, Germany). The 488 nm laser light was used to excite the dynamic fluorescent signals before and after test regents were applied to cells. The Ca^2+^ imaging and recording were carried out at room temperature. The data of imaging traces were representative of 3 separate experiments; 15–20 cells were imaged in each experiment. The imaging data were analyzed with the software LAS-AF-Lite 2.5 (Leica).

### 4.5. Cell Cycle and Apoptosis Assay

Cells were seeded in 6-well plates (Corning, Corning, NY, USA) with 1 × 10^6^ cells per well and treated with NCX blockers. To suppress intracellular Ca^2+^ signal transduction, cells were preloaded with a Ca^2+^ chelator BAPTA-AM (10 μM) for 2 h in advance. After treatment, cells were harvested, washed with cold sterile PBS and fixed with 70% ethanol for 30 min at 4 °C for at least 1 h. For propidium iodide (PI) staining, cells were incubated with ribonuclease A (RNase; 100 μg mL^−1^) for 30 min. PI (10μg mL^−1^) was added and stained cells were kept in the dark at 4 °C until analysis. FACS (Fluorescence activated Cell Sorting) analysis was performed using the Coulter CytoFlexS cytometer (Beckman Coulter, Brea, CA, USA) and the percentage of cells in the G0/G1, S and G2/M phases was determined by ModFit LT 5.0 (Verity Software House, Topsham, ME, USA). To do apoptosis assay, cells were harvested and washed twice with cold PBS and suspended at a density of 1 × 10^6^ cells mL^−1^ in 100 µL of binding buffer containing 5 µL of Annexin V-FITC and 5 μL of PI working solution (100 μg mL^−1^). Apoptosis was analyzed by Coulter CytoFlexS flow cytometer (Beckman Coulter) for at least 10,000 events, and data were analyzed with CyExpert 2.0 (Beckman Coulter).

### 4.6. Western Blot Analysis

Cells were collected and lysed in RIPA buffer (Beyotime, Nanjing, China) supplemented with protease inhibitor (Beyotime). The total protein concentration of each cell sample was measured with the BCA Protein Assay Kit (Sangon Biotech, Shanghai, China). Equal protein extracts were separated by SDS-PAGE and electrophoretically transferred to polyvinylidene difluoride membranes (Millipore, Bedford, MA, USA). The membranes were firstly incubated with anti-NCX1, NCX2, and NCX3 antibodies at 4 °C overnight, then incubated with IRDye 680 LT fluorescent secondary antibody (LI-COR Biosciences, Lincoln, NE, USA). Protein bands were visualized by using the Odyssey Fc Imaging System (LI-COR Biosciences, Lincoln, NE, USA).

### 4.7. Data Calculation and Statistical Analysis

Data analysis was performed by a researcher who was blinded to group assignment. Data were expressed as mean ± SEM and analyzed by GraphPad Prism 7 software (La Jolla, CA, USA). The concentration of the NCX inhibitors yielding half-maximal inhibition (IC_50_) of cell viability was calculated by the equation: viability (% of control) = Bottom + (Top-Bottom)/(1 + 10^((LogIC_50_-*C*)*n)), where *C* is the logarithm of inhibitor concentration and *n* is the Hill coefficient. The difference between two independent groups was analyzed by the unpaired parametric Student’s *t*-test. Data of more than two groups was assessed by the parametric one-way ANOVA followed by a Tukey’s post hoc test. Post hoc tests were conducted when the *F* value achieved the necessary level (*p* < 0.05) and there was no significant variance inhomogeneity. Differences were considered to be significant when the value of *p* < 0.05.

## Figures and Tables

**Table 1:** The half-maximum inhibitory concentration values (IC_50_, μM) of NCX blockers on viability of BRAF mutated melanoma cells.

NCX Inhibitors	Cell Types		
	A2058	A375	C8161
Bepridil	17.10 ± 0.90	16.93 ± 0.96	18.24 ± 0.72
KB-R7943	20.30 ± 2.53	23.31 ± 2.04	15.94 ± 0.60
CB-DMB	3.61 ± 0.43	3.98 ± 0.22	6.55 ± 0.74
SN-6	—	—	—
YM-244769	—	—	—

Note: Each data is represented as mean ± SEM, *n* = 4–5 tests per group.

## Data Availability

The data supporting the findings of this study are available within the article and from the corresponding author upon request.

## Supplementary Materials

The following supporting information can be downloaded at: https://www.mdpi.com/article/10.3390/ijms23020901/s1.
