# Supplementary material for: Suppressing Effect of Na+/Ca2+ Exchanger (NCX) Inhibitors on the Growth of Melanoma Cells
Source: Int J Mol Sci. 2022 Jan 14;23(2):901. doi: 10.3390/ijms23020901 (PMC8780355; doi:10.3390/ijms23020901)
Supplement: Supplementary file 1 [file ijms-23-00901-s001.zip › ijms-1490960-supplementary.pdf]

Supplementary materials

Suppressing effect of Na^+/Ca^{2+} exchanger (NCX) inhibitors on the growth of melanoma cells

Zi-Kai Liu [#], Qing Cheng [#], Xiao-Li Ma and Mingke Song *

Department of Pharmacology and Chemical Biology, Institute of Medical Sciences, Shanghai Jiao Tong University School of Medicine, 280 South Chongqing Road, Shanghai, 200025, China

* Correspondence: Corresponding author: Mingke Song, PhD; Associate Professor; mksong@sjtu.edu.cn

[#] These authors contributed equally to this work.

This PDF document contains:

Supplementary Figure S1
Supplementary Figure S2
Supplementary Figure S3

S1. Three-dimensional (3D) cell culture and treatment

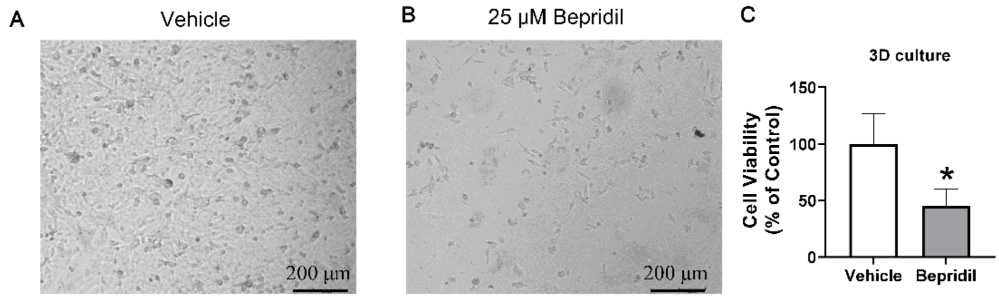

Figure S1. 3D culture of melanoma A2058 cells and treatment with bepridil. (**A-B**) A2058 cells were cultured in a hydrogel-based 3D cell culture platform and treated with vehicle (DMSO, 1/1000, v/v) or bepridil (25 μM) for 48 hours. (**C**) Viability of A2058 cells were reduced by bepridil to 50% of vehicle-treated control level. *$P < 0.05$, bepridil treated groups vs. vehicle treatment, unpaired Student's *t*-test; n = 3 independent experiments.

S2. Western blotting data

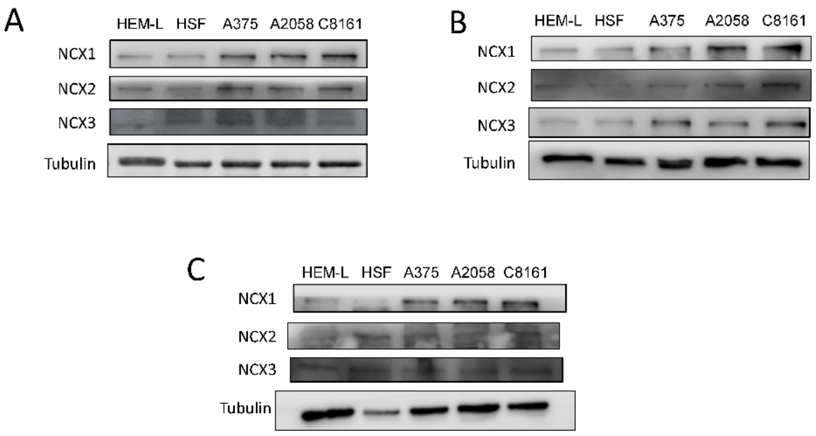

Figure S2. Western blotting analysis of NCX1, NCX2 and NCX3 isoforms in HEM-L, HSF, A375, A2058 and C8161 cell lines. (**A-C**) Three times of independent western blotting results.

S3. Western blotting analysis and cell viability test

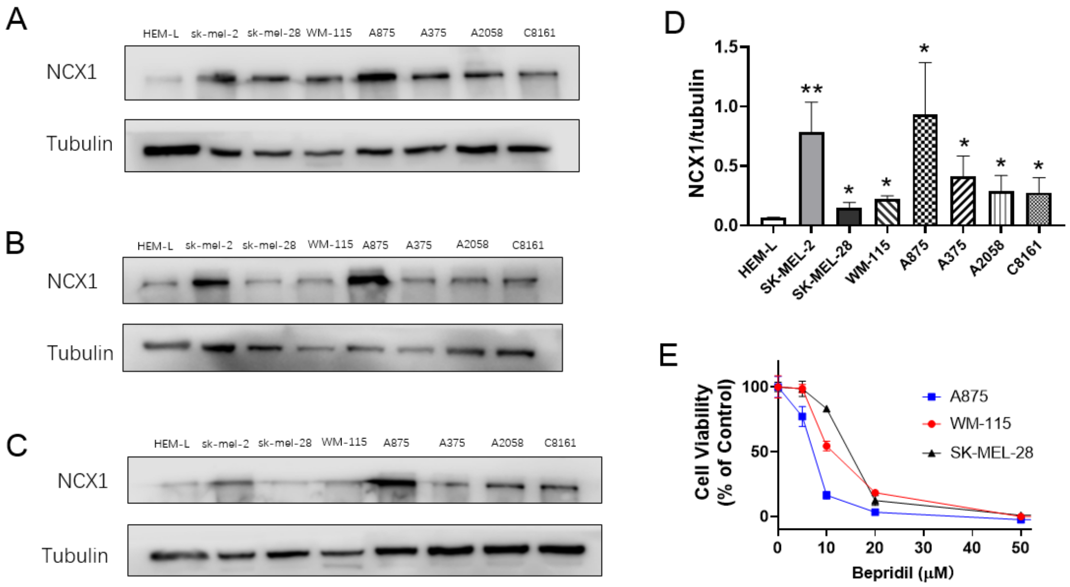

Figure S3. Western blotting analysis of samples from eight different cell lines. (**A-C**) The NCX1 isoform expressed in human epidermal melanocytes-light (HEM-L) and human melanoma cell lines SK-MEL-2, SK-MEL-28, WM-115, A875, A375, A2058 and C8161. (**D**) Quantification of the NCX1 obtained by western blotting. *$P < 0.05$, **$P < 0.01$, each cell line compared with HEM-L; one-way ANOVA followed by a Tukey's post-hoc test, n=3. (**E**) Viability of A875, WM-115 and SK-MEL-28 cell lines after exposed to the NCX blocker bepridil for 48 hours, n = 3 independent experiments for each cell line.
